# Rifampicin-warfarin interaction leading to macroscopic hematuria: a case report and review of the literature

**DOI:** 10.1186/2050-6511-14-27

**Published:** 2013-05-04

**Authors:** Maria AP Martins, Adriano MM Reis, Mariana F Sales, Vandack Nobre, Daniel D Ribeiro, Manoel OC Rocha, Antônio LP Ribeiro

**Affiliations:** 1 Faculdade de Farmácia, Universidade Federal de Minas Gerais, Av. Antônio Carlos, 6627, Pampulha, Belo Horizonte, CEP 31270-901, Brazil; 2 Hospital das Clínicas, Universidade Federal de Minas Gerais, Av. Prof. Alfredo Balena, 110, Santa Efigênia, Belo Horizonte, CEP 30130-100, Brazil; 3 Faculdade de Medicina, Universidade Federal de Minas Gerais, Av. Prof. Alfredo Balena, 190, Santa Efigênia, Belo Horizonte, CEP 30130-100, Brazil

**Keywords:** Tuberculosis, Atrial fibrillation, Rifampicin, Warfarin, Drug therapy, Drug-drug interactions, Hemorrhage, International normalized ratio

## Abstract

**Background:**

Rifampicin remains one of the first-line drugs used in tuberculosis therapy. This drug´s potential to induce the hepatic cytochrome P450 oxidative enzyme system increases the risk of drug-drug interactions. Thus, although the presence of comorbidities typically necessitates the use of multiple drugs, the co-administration of rifampicin and warfarin may lead to adverse drug events. We report a bleeding episode after termination of the co-administration of rifampicin and warfarin and detail the challenges related to international normalized ratio (INR) monitoring.

**Case presentation:**

A 59-year-old Brazilian woman chronically treated with warfarin for atrial fibrillation (therapeutic INR range: 2.0-3.0) was referred to a multidisciplinary anticoagulation clinic at a university hospital. She showed anticoagulation resistance at the beginning of rifampicin therapy, as demonstrated by repeated subtherapeutic INR values. Three months of sequential increases in the warfarin dosage were necessary to reach a therapeutic INR, and frequent visits to the anticoagulation clinic were needed to educate the patient about her pharmacotherapy and to perform the warfarin dosage adjustments. The warfarin dosage also had to be doubled at the beginning of rifampicin therapy. However, four weeks after rifampicin discontinuation, an excessively high INR was observed (7.22), with three-day macroscopic hematuria and the need for an immediate reduction in the warfarin dosage. A therapeutic and stable INR was eventually attained at 50% of the warfarin dosage used by the patient during tuberculosis therapy.

**Conclusions:**

The present case exemplifies the influence of rifampicin therapy on warfarin dosage requirements and the increased risk of bleeding after rifampicin discontinuation. Additionally, this case highlights the need for warfarin weekly monitoring after stopping rifampicin until the maintenance dose of warfarin has decreased to the amount administered before rifampicin use. In particular, patients with cardiovascular diseases and active tuberculosis represent a group with a substantial risk of drug-drug interactions. Learning how to predict and monitor drug-drug interactions may help reduce the incidence of clinically significant adverse drug events.

## Background

Rifampicin remains one of the first-line drugs used to treat tuberculosis (TB) [1,2]. This drug´s use is increasingly common due to the frequency of coinfection with TB and human immunodeficiency virus (HIV) [3,4] and the spread of TB within vulnerable populations [5,6]. Comorbidities and polypharmacy, which are particularly prevalent in older adults, are associated with higher risk of drug-drug interactions [7]. Rifampicin is also a potent inducer of the hepatic cytochrome P450 (CYP) oxidative enzyme system and the P-glycoprotein transport system [8]. The acceleration of drug clearance related to CYP induction by rifampicin may compromise the therapeutic efficacy of several drugs. There are several well-documented examples of clinically significant drug-drug interactions during rifampicin therapy, including interactions between warfarin and rifampicin [8,9].

Warfarin is a coumarin derivative that is widely used to prevent and treat thromboembolic disorders. Racemic warfarin accumulates in the liver, in which both the R and the S enantiomers are metabolically transformed by different pathways [10]. The S enantiomer is approximately 90% oxidatively metabolized, primarily by the CYP2C9 enzyme of the CYP system and, to a lesser extent, by CYP3A4. The less potent R enantiomer is approximately 60% oxidatively metabolized, primarily by the two CYP enzymes CYP1A2 and CYP3A4 and, to a lesser extent, by CYP2C19 [11].

Rifampicin is known to reduce the effect of warfarin on prothrombin activity. This drug-drug interaction has been described in individual case reports [12-18] and pharmacokinetic studies [19-21]. The effect of the rifampicin-warfarin interaction on the anticoagulant response can be tested using the international normalized ratio (INR), which is the current standard for monitoring warfarin responses. Patients with sub- and supratherapeutic INR values are at a higher risk of clinical complications [11]. However, there are no published long-term follow-up studies focused on bleeding events and INR values after termination of the co-administration of rifampicin and warfarin.

In this report, we describe a 35-month follow-up of a patient chronically treated with warfarin for atrial fibrillation (AF) and undergoing concomitant 6-month TB therapy. Warfarin resistance was observed at the beginning of the TB treatment, and a continuous dosage increase was required to achieve and maintain INR control. Following rifampicin discontinuation, the patient was diagnosed with macroscopic hematuria, and the warfarin dosage was gradually decreased to stabilize the INR within the therapeutic range. Here, we also review studies focusing on the rifampicin-warfarin interaction.

## Case presentation

A 59-year-old, non-white woman was diagnosed with AF in May 2009, when warfarin therapy was initiated with a therapeutic INR range of 2.0-3.0. She was referred to the anticoagulation clinic of a university hospital in March 2010. Her typical warfarin dosage was nearly 52.5 mg/week. The patient’s medical history included systemic arterial hypertension, pulmonary arterial hypertension, rheumatic cardiopathy, asthma and chronic emphysema. She was also subjected to a biological mitral valve replacement in 2006. The patient denied any alcohol consumption and showed no evidence of hepatic or renal dysfunction. Her drug list included ferrous sulfate, hydrochlorothiazide and enalapril.

By the time the patient was sent for outpatient anticoagulation control, she had been diagnosed with pleural TB and had begun treatment with isoniazid (400 mg/day), rifampicin (600 mg/day) and pyrazinamide (2 g/day). The pyrazinamide was discontinued after two months. Subtherapeutic INR values were obtained for several weeks after initiating TB therapy. Routine reevaluations were necessary for INR monitoring and warfarin dosage adjustments. Thus, the warfarin dosage was gradually increased from 45 mg/week to 80 mg/week. Three months were necessary to reach a stable INR, which was maintained for an additional three months.

**Figure 1:**
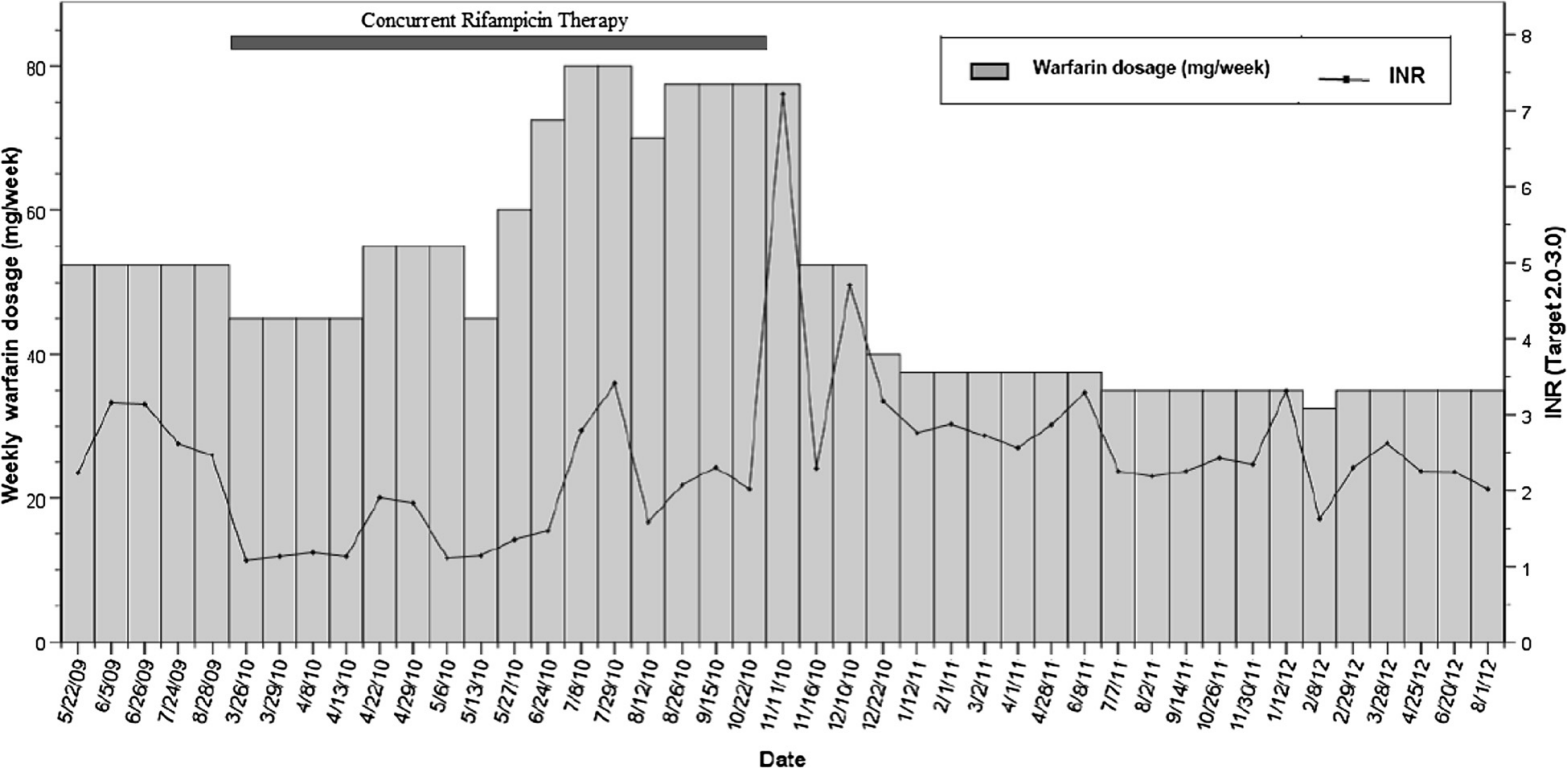
**Weekly warfarin dosage, INR values, and concurrent rifampin therapy over time.** The x-axis represents time in relation to the dates of the patient’s anticoagulation clinic appointments. The left y-axis represents the prescribed warfarin dosage in milligrams/week, which is shown by the vertical bars. The right y-axis represents the INR values, which are depicted by the black points (therapeutic range: 2.0-3.0). The time period of the concurrent rifampin therapy is shown as a horizontal dark bar.

On October 15th, 2010, the patient´s rifampicin and isoniazid use was discontinued by an infectious disease specialist. One week later, a therapeutic INR of 2.02 was obtained. On November 11th, 2010, the patient noticed significant macroscopic hematuria that lasted for three days and a supratherapeutic INR (7.22) was measured on the same day. The warfarin doses were interrupted for two days, and the weekly dose was reduced from 77.5 mg to 52.5 mg (33%), according to the hospital protocol. Vitamin K or fresh frozen plasma was not administered due to the absence of hemodynamic instability or another reason justifying immediate INR reduction. Five days after the dose reduction, the patient´s INR was 2.29. The patient had no history of bleedings, and there were no reported dosing errors or changes in drug therapy, vitamin K intake or medical status. A urinalysis performed five days after the first bleeding episode showed no significant presence of erythrocytes. Afterward, INR control was reached at a weekly warfarin dose of 37.5 mg. Sequential INR values and related warfarin weekly doses for the 35-month follow-up are presented in Figure 1.

## Discussion

In this case report, a patient chronically treated with warfarin showed anticoagulation resistance at the beginning of rifampicin therapy, as demonstrated by repeated subtherapeutic INR values. During this phase, there was an augmented risk of cardioembolic events. Three months of sequential increases in the warfarin dosage were necessary to reach a therapeutic INR. A hyperanticoagulation state occurred after discontinuation of rifampicin, resulting in macroscopic hematuria and the need for an immediate reduction in the warfarin dosage. The patient´s INR was still within the therapeutic range one week after withdrawing rifampicin. However, a supratherapeutic INR value and bleeding were observed four weeks after the interruption of rifampicin therapy, suggesting a delayed offset of the rifampicin-warfarin interaction. The evaluation of the present case was based on the criteria for adverse event reports proposed by Kelly [22]. The Naranjo algorithm [23] and the Drug Interaction Probability Scale (DIPS) [24] were also employed to evaluate the causality of the adverse event. The effect of each medication on the warfarin anticoagulant response was considered. Rifampicin showed a score of 8 (probable) for both methods, which was the highest score obtained for the medications in use.

The proposed mechanism for the rifampicin-warfarin interaction involves the induction of the isoenzymes CYP2C9, CYP3A4, CYP1A2 and CYP2C19 [25,26], accelerating the clearance of both the R and the S enantiomers of warfarin. Enzyme induction typically exhibits a slow onset and long-term recovery time. In particular, CYP induction depends on the synthesis of new drug-metabolizing enzymes, with the initial effects detectable within the first two days of concurrent therapy. However, it generally takes at least one week to observe the effects of maximal induction. The onset of CYP stimulation is also dependent on the half-life of the inducer. As rifampicin exhibits a relatively short half-life, steady-state serum concentrations are obtained faster when rifampicin is compared with other inducing drugs with longer half-lives [26,27]. The dissipation of CYP induction after the discontinuation of rifampicin occurs gradually, depending on the drug´s elimination and the gradual decay of the enhanced enzymatic activity in the liver [8,26]. The concurrent use of isoniazid may cause an opposite effect on the liver by inhibiting CYP3A4 [28-30], leading to the accumulation of the less potent R enantiomer. As an additional mechanism, an acquired inhibition of fibrin stabilization has been associated with isoniazid therapy [31]. In the present case, the stimulatory effect of rifampicin on the liver seemed to be clinically predominant over the effect of the concomitant use of isoniazid on the coagulation state, which is consistent with previous findings [12,14,15].

The prediction of a patient’s response to warfarin and of the precise magnitude of the dosage adjustments required when rifampicin is initiated or discontinued is challenging. In the case presented here, it was necessary to double the warfarin dose at the beginning of rifampicin therapy and to reduce the warfarin dose by approximately 50% after discontinuing rifampicin to attain a therapeutic INR. Other recent case reports have demonstrated an increased sensitivity to warfarin after stopping rifampicin therapy and the need for more than four weeks to achieve a therapeutic INR, until the drug-drug interaction dissipated [16-18]. However, supratherapeutic INR values were not associated with bleeding in these studies (Table 1).

**Table 1:** Summary of recent studies reporting drug-drug interactions involving rifampicin and warfarin in humans

**Study**	**Country**	**Type of study**	**Number of patients**	**Sex**	**Age (years)**	**Warfarin administration**			**Comments**
						**Chronic dose**	**Titration**	**Plasma levels**	
Lee & Thrasher, 2001 [16]	USA	Case report	1	Male	58	Yes	Yes	No	A 233% increase in the warfarin dose over four months could not attain a therapeutic INR^*^ during the use of rifampicin. A therapeutic INR^*^ was obtained after a 70% reduction in the warfarin dose over four to five weeks after rifampicin discontinuation.
Kim *et al.*, 2007 [18]	USA	Case report	1	Male	79	Yes	Yes	No	Increases of 500-600% in the warfarin dose were insufficient to maintain an INR^*^ in the therapeutic range. After rifampicin was discontinued, the warfarin dose was gradually reduced over two months to achieve a therapeutic INR^*^.
Krajewski, 2010 [17]	USA	Case report	1	Male	71	Yes	Yes	No	The warfarin dose was sequentially increased by up to 500%, from a starting point of 35–40 mg/week to an endpoint of 175 mg/week over two months, with no achievement of a therapeutic INR^*^. After rifampicin discontinuation, the warfarin dose was gradually reduced to the initial regimen over three to four months to attain a therapeutic INR^*^.

* *INR* international normalized ratio.

The potential of rifampicin to induce warfarin metabolism has been well known for decades, although only a limited number of case reports have been published. In the current report, long-term INR monitoring helped demonstrate the effect that the concomitant use of rifampicin may have on the warfarin anticoagulant response and on the risk of adverse events, such as bleeding. Our report serves to forewarn health professionals that a patient should be monitored weekly after stopping rifampicin, until the maintenance dose of warfarin has decreased to the dose administered before the use of rifampicin. Although our findings cannot be generalized, this case report illustrates the increased risk of drug-drug interactions in patients with comorbidities and complex dosing regimens. Close monitoring and patient education may help improve the quality of care delivered to these patients.

## Conclusions

This case demonstrated the influence of rifampicin therapy on warfarin dose requirements and the increased risk of bleeding. The INR should be monitored weekly until a new stable dose is achieved after rifampicin discontinuation to prevent clinical complications related to unstable INR values. In particular, patients with cardiovascular diseases and active TB represent a group with a substantial risk of drug-drug interactions. Learning how to predict and monitor drug-drug interactions may help reduce the incidence of clinically significant adverse drug events.

### Consent

Written informed consent was obtained from the patient for publication of this case report.

## Competing interests

The authors declare that they have no competing interests.

## Authors’ contributions

MAPM and MFS were directly involved in the patient’s follow-up and data acquisition. DDR advised on the management of the patient and helped with data interpretation. MAPM drafted the manuscript. AMMR and VN assisted in editing the manuscript. MOC and ALPR, who headed the medical team, made substantial contributions to the conception, design and revision of the study. All of the authors read and approved the final manuscript.

## Pre-publication history

The pre-publication history for this paper can be accessed here:

http://www.biomedcentral.com/2050-6511/14/27/prepub
